# Special Issue: Molecular Properties and the Applications of Peptide Nucleic Acids

**DOI:** 10.3390/molecules23081977

**Published:** 2018-08-08

**Authors:** Roberto Corradini

**Affiliations:** Department of Chemistry, Life Sciences and Environmental Sustainability, University of Parma, Parco Area delle Scienze 17/A, I-43124 Parma, Italy; roberto.corradini@unipr.it

Polyamide analogs of DNA, or peptide nucleic acid (PNA), were first proposed in 1991 by a group of chemists and biochemists in a memorable *Science* paper [1]. At that time, it was astonishing that such a dramatic change in the structure of nucleic acids, i.e., subsituting the sugar–phosphate backbone with a polyamide constituted of repeated *N*-(2-aminoethyl)glycine units, could give rise to molecules that not only behaved like DNA, but also showed improved binding propeties and improved selectivity. Since then, PNA has attracted the interest of a growing number of scientists in several fields [2]. This Special Issue provides a good overview of these various fields and of the vitality of the research into these molecules.

In synthetic chemistry, a large effort has been dedicated to creating new DNA/RNA binding molecules using PNA as a model system or as a scaffold for further elaboration [3,4]. This is the case in the paper by Elksens et al. [5], where PNA was decorated with furan moieties to obtain interstrand cross-linking (ICL); the use of backbone-modified PNAs in this case improved the recognition properties and thus the reaction outcome. A series of papers in this Special Issue also report the use of modified PNAs [6,7,8]. Some of these were designed over the years in order to show better solubility and better binding abilities, others were designed for the decoration of PNAs with functional moieties; these can bring new properties and thus new applications. Exploiting the partial formation of triplexes has, from the beginning of PNA history, been one of the most intriguing and unique properties of PNAs, allowing DNA strand invasion; this has been exploited in the tail-clamp design of PNA by providing a homopyriimidine tail to a long mixed-sequence PNA. The paper by Kunihiro Kaihatsu and coworkers reports further detail in this field, showing the effect of azobenzene linkers of tail-clamp PNAs on their DNA binidng ability [9].

In diagnostics, PNA has been established among the best-performing probes for DNA recogniton, and its unique properties allow highly selective analysis to be performed, in particular point mutation and the detection of single nucleotide polymorphisms (SNPs). This analysis can also be performed with high sensitivity, allowing for ultrasensitive and PCR-free analysis. The review by Spoto and collaborators provides an updated view of the approaches using PNA probes for RNA and DNA detection in analytical chemistry for cancer diagnosis [10]. Two specific papers demonstrate the usefulness of PNA in the detection of rare mutations (Chiuan-Chian Chiou and co-workers) [11] and in the assessment of the authenticity of medicinal plants (Byeong Cheol Moon and co-workers) [12]. The use of PNAprobes has been further extended to the detection of DNA/RNA in cells and tissues; the paper by Bruce Armitage and collaborators describes an interesting application, using PNA miniprobes for telomer length measurement [6]. This field is ready for a massive technology transfer in the rapidly advancing sector of sensors and diagnostic kits, not only for biomedical applications, but also for other sectors such as the rapid screening of the identity of biological items and materials (e.g., food and raw material control).

As tools in molecular biology and medicine, PNA has enabled the design of new methods for gene regulation by acting at the transcription (anti-gene), splicing and translation (antisense), or microRNA (anti-miR) levels. In particular, these approaches can be useful for some diseases that do not currently have efficient pharmacological treatments, as illustrated by the two papers dealing with the regulation of cystic fibrosis processes. Giorgia Oliviero and coworkers report using PNAs in a ‘masking’ approach to limit microRNA activity [13], while Roberto Gambari and collaborators show the possibility of increasing cystic fibrosis conductance regulator (CFTR) levels by acting with anti-miR PNAs [14]. Thus, new perspectives are arising for the treatment of this severe and debilitating disease.

The appropriate tailoring of the PNA structure has provided new solutions and new applications in molecular biology, based on the reactions induced by the PNA on its target. The review by Makoto Komiyama and collaborators describes the use of PNA as an inducer of DNA cutting for gene repair and gene editing, which allows the manipulation of DNA and overcomes the limitations of the sequences presented by restriciton enzymes [8]. The paper by Roger Stromberg and co-workers illustrates the possibility of inducing hydrolytic cleavage in target RNA, thus creating precise, artificial, RNAse-like activity [7].

Gene editing techniques have become a field of major interest in biology and medicine, and an increasing number of applications for these can be envisaged in the near future. In addition to Clustered Regularly Interspaced Short Palindromic Repeats (CRISPR)/CRISPR-associated protein 9 (Cas9), Transcription activator-like effector nucleases (TALEN), and zinc finger nuclease techniques, PNA-based approaches have also been described. The very interesting and up-to-date review by Peter Glazer and co-workers describes very well how these molecules can outperform the present standard methods in terms of precision, showing reduced off-target effects [15]. This field is certainly one of the most promising for the future of PNA applications.

Finally, although still rarely considered, PNA can be a component of the so-called ‘DNA nanotechnology’, and is especially suitable due to its chemical robustness and properties of self-assembly and hetero-association with natural nucleic acids. Nanoassembly, molecular machines, switches and logic gates can be designed using PNAs as components or as switching stimuli. A good example of this is reported by Kenji Usui and co-workers in this issue, where a DNA G-quadruplex secondary structure and nanostructural shape were shown to be affected by PNA switching segments [16].

Thus, we can conclude that the present issue of *Molecules* shows how PNA technology is still improving and that new applications and new knowledge is still arising from the study of this class of molecules, which has been researched for 27 years but has still not been fully explored.
